# Advances in Research on the Protective Mechanisms of Traditional Chinese Medicine (TCM) in Islet *β* Cells

**DOI:** 10.1155/2019/7526098

**Published:** 2019-08-21

**Authors:** Na Li, Hongli Zhang, Xiaohua Li

**Affiliations:** Department of Endocrinology, Seventh People's Hospital Affiliated to Shanghai University of TCM, Shanghai 200137, China

## Abstract

The dysfunction and decreased number of islet *β* cells are central to the main pathogenesis of diabetes. Improving islet *β* cell function and increasing the number of *β* cells are effective approaches to treat diabetes and constitute the main direction of diabetes drug development. The role of Chinese medicine in the treatment of diabetes began to be recognized. In recent years, Chinese medicine monomers have been found to increase insulin synthesis and secretion, reduce *β* cell-apoptosis, and protect the function of *β* cells. The results of in vivo animal experiments and in vitro studies on insulinoma cells also suggested TCMs could promote the proliferation of pancreatic islet *β* cells and induce other cells differentiation or transdifferentiation to islet *β* cells. Thereby, they may play a role in the treatment of diabetes. In this paper, we will review islet *β* cell protection with TCMs and the related mechanisms found in recent studies. An in-depth explanation of the role of TCM in islet *β* cell protection can provide a theoretical basis and research ideas for the development of TCM-based diabetes treatment drugs.

## 1. Introduction

According to the WHO, the number of patients with type 2 diabetes mellitus (T2DM) will reach 300 million by 2025. Islet *β* cell function failure is the core principle of diabetes pathogenesis and the pathophysiological basis for the occurrence and development of the disease. Insulin function failure include decrease in the number of islet *β* cells, defect of insulin secretion and synthesis of mature insulin. In traditional Chinese medical science, diabetes is called consumptive thirst (XIAO KE). T2DM is categorized as “Xiao Ke” in TCM mentioned in “Huang Di Nei Jing Su Wen” (Pre-Qin Dynasty to Han Dynasty). TCM theorizes that the internal organ weakness, excessive intake of fat and sugar, and mood disorders are the main causes of “Xiao Ke.” The pathogenesis involves an insufficiency of Yin and excessive dryness in vivo, and its governance involves clearing the lungs, moistening dryness, and invigorating the lungs and kidneys. The key etiology of diabetes mellitus included phlegm, heat, dryness, and blood stasis, and the diseased organs were the spleen, lung, stomach, and kidney. Therefore, Chinese medicine often treats diabetes by Triple-jiao deficiencies argument. Diabetes is classified into three types in Chinese medicine including damp-heat trapping spleen, gastrointestinal heat retention, and dry-heat damaging Yin. At present, it is believed that islet *β* cell damage is mainly caused by qi, blood, and a Yin Yang deficiency, complicated by damp-turbid phlegm, heat, and heat retention. As a complementary and alternative medicine, traditional Chinese medicine (TCM), which has been practiced for thousands of years, has been shown to possess satisfactory clinical effectiveness for diabetes mellitus and its complications, using medications such as *Gegen Qinlian Decoction* [1], *JinQi Jiangtang* (*JQJT*) [2], and *Xiaokeping* (*XKP*) [3]. *Pueraria* (*Ge Gen*), *Astragali* (*Huang Qi*), *ginseng* (*Ren Sheng*), *digitalis* (*Di Huang*), *Anemarrhena* (*Zhi Mu*), *Berberine* (*Ber*) (*Huang Lian*), *rhubarb* (*Da Huang*), and other TCMs often have a hypoglycemic effect. There are currently more than 100 types of Chinese medicines that treat diabetes. *Huangqi* (“Gan” flavor) Bunge and *Huanglian* (“Ku” flavor) are some of the most frequently prescribed herbs [4]. At present, the active ingredients of TCMs, such as alkaloids, polysaccharides, flavonoids, terpenoids, and saponins, are widely studied [5]. This paper aims to summarize the mechanisms of TCM that protect *β* cells and the prospects for future research.

## 2. Improvement in Insulin Secretion in Islet *β* Cells

### 2.1. Promotion of Insulin Synthesis and Secretion from Islet *β* Cells

In both type 1 (T1D) and type 2 diabetes (T2D), the deterioration of glycemic control over time is primarily caused by an inadequate mass and progressive dysfunction of *β* cell, leading to the impaired insulin secretion. The beta cell senses an elevated level of glucose in the plasma by glucokinase. Glucose enters the tricarboxylic acid cycle (TCA) through solute carrier family 2 (facilitated glucose transporter), resulting in the productin of NADH and FADH2 which promote the ATP production. The rise of the ATP : ADP ratio in the cytoplasm closes the plasmalemmal ATP-sensitive potassium channel (KATP). This results in an influx of Ca^2+^ into the cell that triggers the exocytosis of insulin granules [6].

Studies have shown that *ginsenosides* can regulate islet *β* cell mitochondrial function, increase ATP content, reduce UCP2 content, and increase insulin synthesis and secretion. *Ginsenoside Rb2* [7](0.5 *μ*g/mL) can promote insulin synthesis, and *ginsenoside Rg3* [8](10 *μ*M) increases insulin secretion by INS-1 cells in a high-glucose environment by 47%. *Guavenoic acid* (*GA*) [9] (1 nmol/L) promotes the synthesis and secretion of insulin, possibly via downregulation of PTP1b mRNA and upregulation of the PPAR-*γ* and PDX-1 genes. Guava leaf extract (GLE) [10] may improve the ability of islet *β* cells to secrete insulin and may ameliorate oxidative stress in islet *β* cells.

*Berberine* [11] can increase the expression of the HNF-1*α*/PDX-1 pathway and insulin secretion, and this effect becomes larger with increasing concentrations. *Baicalein* (*Huangqin*) [12] regulates insulin secretion by inhibiting regulation of the intracellular Ca^2+^ concentration in islet *β* cells by voltage-dependent K^+^ channels in the cell membrane. *Resveratrol* and *curcumin* [13] can regulate the expression and activity of phosphodiesterases (PDEs), increase the level of cAMP, and stimulate insulin secretion. *Syzygium cumini* (*L*.)–(*Myrtaceae*) [14] stimulates and modulates insulin release from islet *β* cells.

### 2.2. Promotion of Proinsulin Cleavage into Insulin

Proinsulin is an insulin precursor synthesized in the endoplasmic reticulum. It is folded into active insulin by modification and shearing by a series of enzymes [15]. The presence of unfolded or misfolded insulin increases the level of nonnaturally folded polypeptide in the endoplasmic reticulum, thereby causing an imbalance of proinsulin homeostasis, an increased proinsulin/insulin ratio, a decrease in active insulin, and islet *β* cell dysfunction [16]. *Lycium barbarum polysaccharides* [17] increase the transcription and expression of proinsulin genes and proteins as well as the transformation of proinsulin into mature insulin.

## 3. Increasing Islet *β* Cell Mass

There emerged a consensus that progressive *β*-cell decline is a characteristic of islet pathology in type 2 diabetes, and restoring an effective *β*-cell mass is a therapy for diabetes. *β* cell mass is dictated by a dynamic balance between neogenesis, proliferation, and apoptosis. Several TCMs have been implicated to promote the proliferation and regeneration of pancreatic islet *β* cells in in vivo animal experiments and in vitro studies on insulinoma cells.

### 3.1. Promotion of *β* Cell Proliferation

Studies have shown that *Salidroside* (*Hongjingtian*) [18] promotes the mRNA expression of insulin, PDX-1, GLP-1R, and other genes, reduces the level of IL-1*β*, and promotes the proliferation of *β* cells. *Cortex Lycii* (*Digupi*) (2 g/L) [19] increased the proliferation rate of islet *β* cells and inhibited the apoptosis of islet *β* cells. *Portulaca oleracea* L. (*POP I*) [20] activates JNK, PDX-1, PPAR*γ*, and ERK, which affects islet *β* cell mitosis and promotes cell proliferation. *Puerarin* [21] can upregulate the expression of PDX-1 and promote the proliferation of islet *β* cells. *Baicalein* [22] can increase islet *β* cell mass in mice. *Baicalein*-treated diabetic mice displayed fewer apoptotic islet *β* cells and increased viability of clonal islet *β* cells and human islet cells.

### 3.2. Induction of Stem Cell Differentiation to Islet *β* Cells

Stem cells, including embryonic and adult stem cells, have the ability to renew and undergo multipotent differentiation. Pancreatic duct epithelial cells have stem cell properties [23]. *Tripterygium wilfordii polysaccharide* (*TWP*) [24] can induce islet stem cells to differentiate into spherical islet cell structures that produce an elongated tubule connected to the extracellular environment through the expression of *β* cell functional proteins. This finding indicates that TWP can induce islet stem cells to differentiate into *β* cell-containing islet cell clusters when cultured in vitro. *Resveratrol* (*Baililu*) [25] induces embryonic stem cells and the pluripotent stem cell line (hiPSC) MSUH-001 to differentiate into cells expressing specific protein markers of islet *β* cell function, such as PDX-1, that have an insulin secretion function. Differentiated cells accumulate in islet-like clusters when transplanted into mice with streptozotocin- (STZ-) induced diabetes. The cells secrete insulin and decrease blood glucose of STZ-treated mice. It indicated that resveratrol can promote stem cell differentiated to islet *β* cell and improves *β* cell maturation. In another study, it has been found *Syzygium cumini* (L.) Skeels SC2 [26] could induce *β* cell neogenesis in islets of STZ-treated mice.

### 3.3. Transformation of Islet *α* Cells into Islet *β* Cells

The epithelium of the pancreas includes *β* cells, duct cells, acinar cells, and *α* cells. When pancreatic damage, metabolic stress, and genetic manipulation occur, other pancreatic epithelial cells may be induced to transdifferentiate into *β* cells, which may then be induced to differentiate or dedifferentiate into immature *β* cells [27]. Islet *α* and *β* cells are derived from the same progenitor cells and express homologous proteins that sense blood glucose levels and promote hormone release, but *α* cells have the opposite effect on regulating blood glucose levels. Studies have shown that when the Pax4 gene is ectopically expressed, *α* cells are induced to differentiate into *β* cells [28]. Pax4 and Arx are the main regulatory genes that induce the transformation of *α* cells into *β* cells, and Arx represents the main trigger of *α* cell-mediated *β*-like cell regeneration. *Artemisinin* (*Qinghao*) [29] increases GABA signaling, and artemether induces ARX transport from the nucleus to the cytoplasm, eliminates ARX from chromatin, and prevents *α* cells from secreting glucagon. The *α* cells acquire the characteristics of *β* cell and then transform into mature *β* cells with insulin-secreting function.

### 3.4. Reduction of *β* Cell Apoptosis

Apoptosis plays important roles in the pathophysiology of T2DM, which resulted in defective insulin secretion and loss of *β*-cell mass. *β*-cell apoptosis is mediated through a milliard of caspase family cascade machinery in T2DM. Hyperglycemia-induced *β*-cell apoptosis has been extensively studied on the balance of proapoptotic Bcl-2 proteins (Bad, Bid, Bik, and Bax) and antiapoptotic Bcl family (Bcl-2 and Bcl-xL) toward apoptosis in vitro isolated islets and insulinoma cell culture. Many pathways, for example, PI3K/AKT, MAPK, and autophagy, involved in apoptosis. There are several kinds of TCMs for treating T2DM, and their mechanisms to lower blood glucose levels include reduction of *β* cell apoptosis.

*Ginsenoside Rg3* [30] can inhibit *β* cell apoptosis through activating phosphorylation of ERK and p38 MAPK or regulating P44/42 MAPK activation in the *β* cells. *Red ginseng* [31] can inhibit the expression of bcl-2, reduce the level of Bax and the activity of caspase-3, reduce the production of proinflammatory cytokines, and inhibit cell apoptosis in *β* cells of CsA-induced injury. *Panax notoginseng saponins* (*PNS*) [32] can upregulate the expression of mir-181b and caspase-3 and increase the expression of bcl-2 and activation of the PI3K-AKT-mTOR pathway, which blocks the expression of the autophagy gene beclin-1 and the autophagy membrane marker LC3-II, thereby reducing apoptosis and autophagy. *Berberine* [11] can reduce the expression of PTEN, activate the PI3K/Akt signaling pathway, decrease the expression of caspase-3, and significantly reduce the rate of apoptosis. *Puerarin* [21] and *Cortex Mori Radicis with drug serum* [33] may downregulate caspase-3 expression, upregulate bcl-2, downregulate Bax expression, and inhibit islet cell apoptosis through the PI3K/Akt pathway. *Ophiopogonis Radix* [34] protects islet *β* cells by inhibiting the expression of NF-*κ*B to reduce apoptosis. The *Jin Li Da* (*JLD*) granules contain over a dozen Chinese medicinal herbs (CHM) [35], including *ginseng* (*Renshen*), *puerarin* (*Gegen*), *Coptis chinensis* (*Huanglian*), and *Radix polygonati officinalis* (*Yuzhu*). *JLD* might decrease *β* cell apoptosis through AMP-activated protein kinase activation.

### 3.5. Maintenance of Islet Cell Structure Integrity

TCMs can protect and repair the structural integrity of islet *β* cells. The number of islet and endocrine cells in a diabetic model treated with total flavonoids *of Lycium barbarum* (*TFLB*) [36] was increased compared to that of the control group, and the islet cell volume was slightly increased. In addition, the number of vacuolar cells decreased, the cell arrangement was uniform, and the structure and morphology were normal. In *red ginseng-*treated [31] islet cells with CsA-induced injury, the islet size was increased, insulin immunoreactivity was enhanced, islet cell morphology was well preserved, the islet cell boundary was irregular, and vacuolization was reduced. After treatment with *puerarin*, *oleanolic acid*, *and astragalosid*e [37], the islet cells were structurally intact, and the islet cell boundary was clear and similar to the normal structure. In the *ginsenoside Rg1-*treated group [38], the islet cell volume was increased, the areas of islet cells were significantly increased, mitochondria were slightly swollen, the crista were expanded in the endoplasmic reticulum, and Golgi body expansion was obviously reduced. The endocrine *β* cell morphology and the number of secretory granules were similar to those of normal cells, and aging characteristics were not obvious. Administration of *Astragalus* total saponins and curcumin in a ratio of 3 : 7 obviously reduced the pathological changes of pancreatic tissue [39]. *GLE-*treated [10] diabetic rats had islet cells with normal granules and exhibited cell hyperplasticity.

## 4. Reduction of Oxidative Stress (OS) Damage in Islet *β* Cells

Insulin secretion abnormalities, high glucose, fat accumulation toxicity, apoptosis, and inflammatory reactions cause increases in highly active ROS, such as H_2_O_2_ and RNS. Under these conditions, the activity of SOD, CAT, GSH-Px, and other antioxidants is inhibited and the antioxidant enzyme level in islet *β* cells is decreased, which resulted in the defect of repairing DNA after oxidative damage ROS can directly damage islet *β* cells, promote apoptosis, and affect the insulin signal transduction pathway, indirectly inhibiting islet *β* cell function. The flavonoids, polysaccharides, and glycosides in TCMs can enhance antioxidant enzyme activity, reduce peroxide accumulation, block the protein kinase C signaling pathway and cytokine activity, and repair OS damage in islet *β* cells.

*Cordyceps sinensis* (*CS*) [40]can decrease NO and ROS production, reduce Ca^2+^ concentration, and enhance the expression of UCP-2 protein in islet cells damaged by IL-1*β*, effectively scavenge free radicals, and reduce oxidative damage. *Puerarin* [41] reduces the MDA content, increases SOD activity, reduces serum levels of PGE2, ET, H_2_O_2_, and NO, and inhibits the mRNA and protein expression of ICAM-1, NOX2, and NOX4. *Salidroside* [42] can downregulate TXNIP mRNA and upregulate TRX mRNA levels, resulting in low ROS levels. *Total flavonoids of Lycium barbarum* (*TFLB*) [36] can increase SOD and GSH levels and reduce MDA content. *Anoectochilus roxburghii* [43] can remove DPPH and ABTS, thereby reducing NO production, and the expression of iNOS, COX-2, and IL-1*β* reduces OS. *Oleanolic acid* [37] inhibits *α*-glucosidase activity, OS, and the inflammatory reaction. *Cinnamaldehyde* [44] directly increases the antioxidant enzyme activity of islet cells and reduces the production of free radicals. *GA* [45] can inhibit the production of ROS in INS-1 cells and alleviate OS. Many TCMs can exert their biological functions through the JNK signaling pathway to reduce oxidative stress. *GinsenosideRb1* [46] inhibits the JNK signaling pathway, as well as JNK1 and c-Jun expression in STZ-induced diabetic rats, thereby resulting in negative regulation of the expression of the inflammatory molecules IL-6, IL-1*β*, and TNF-*α*, which reduces inflammation and OS damage. *POP I* [20] downregulates the expression of the JNK1, P38, and ERK proteins in the JNK pathway and alleviates the damage induced by oxygen-free radicals. *Crude Portulaca Oleracea* L. *Polysaccharide* (*CPOP*) [47], which is an antioxidant with an antidiabetic and anti-inflammatory effect, reduces MDA content and increases SOD activity in the diabetic rats. *Mogroside* [48] can significantly reduce the ROS content in NIT-1 cells, upregulate the expression of GLUT-2 and the pyruvate kinase gene, inhibit the activation of the JNK signal transduction pathway, and reverse the effect of Foxo1, which slows OS damage and protects islet secretion. *Curcumin* [49, 50] antagonistically binds to TLR-4 and prevents caspase-8 from activating apoptotic DNA fragments, reducing the number of cells affected by proinflammatory cytokines (such as GM-CSF, G-CSF, and IL-6), NK T cells, and macrophage infiltration. Administration of 4–8 g/kg of *Polygonatum odoratum* [51] can reduce interferon IFN-*γ* and IL-4 levels, inhibit inflammatory cell infiltration and the polarization of Th1 cells, and reduce the oxidation of islet *β* cells after stimulation.

## 5. Promotion of GLP-1 Secretion

GLP-1 is a peptide hormone secreted by intestinal L cells and acts on islet *β* cells to promote insulin gene transcription, increase insulin synthesis and secretion, stimulate the proliferation and differentiation of *β* cells, and inhibit *β* cell apoptosis. Several TCMs have been demonstrated to simulate GLP1 secretion. The active ingredients of *Rhizoma coptidis* [52] can increase the secretion of GLP-1. In vivo and in vitro experiments on rats and STC-1 cell lines have demonstrated that *emodin* [53] promotes the secretion of GLP-1 by regulating PPAR*δ* in a dose-dependent manner. The accumulation of *ginsenosideRe* [54]in the intestine inhibits the activity of *α*-glucosidase and promotes the secretion of GLP-1 by L cells as well as the secretion of glucagon.

## 6. Other Functions

The protective and regenerative effects of TCMs on islet *β* cells have been utilized in surgical transplantation. Li et al. found extract of *trichosanthin* [55] could slow reject reaction and protect the transplanted *β* cells when it was applied in transplantation cases. Serum containing *Euonymus fortunei* [56] can increase the insulin and GSH content of islet cells, protect the islet cell secretion function, increase the cell survival rate, and reduce ROS-induced islet cell damage before transplantation. *Gastrodin* (*GAS*) [57] regulates Nrf2, affects mitochondrial dysfunction, and exhibits antioxidant, anti-inflammatory, and antiapoptotic effects in response to redox damage. *Gastrodia elata Blume* (GEB) water extract [58] dose-dependently improved hypothalamic insulin signaling, enhanced systemic insulin sensitivity during a euglycemic hyperinsulinemic clamp, and increased insulin secretion under high glucose stimulation. *Tangminling* (TML) *pills* [59]mainly contain *Rhizoma coptidis*, *Rheum officinale* Baill, *Scutellaria baicalensis* Georgi, and *Bupleurum chinense* DC and might improve *β* cell function in T2DM patients. *Tianmai Xiaoke Pian* (*TMXKP*) [60] is composed of Tianhuafen, Maidong, and Wuweizi and could improve ischemia-reperfusion through the PI3K/AKT pathway.

## 7. Outlook

Type 2 diabetes is characterized by hyperglycaemia due to insulin resistance in peripheral tissues and a defect of *β* cell function. Many factors have been implicated in *β* cell dysfunction in T2DM. Traditional Chinese medicine (TCM) has been implicated in practical use in China for thousands of years and has accumulated substantial front-line experience in treating T2DM. In this review, we have highlighted the mechanism of TCMs in protecting *β* cell function. The current research is mainly focused on improving insulin secretion of *β* cell, promoting islet cell proliferation, reducing OS damage, and inhibiting apoptosis. The list of the TCM and their role in protective *β* cell are summarized in Table 1. It is of note that the effects of TCMs on *β* cell function are mainly evaluated in cellular and rodent models. Whether the effectiveness of improvement of islet *β* cell function of TCM is shown in animal studies remains unclear. Clinical data are required to access their efficacy and side effects on patients in the future.

In summary, though great efforts are still needed to better understand its mechanisms and clinical relevance, TCM possesses great potential as a vast and readily available source for finding and developing new drugs to treat T2DM through improving *β* cells function. With the high prevalence of T2DM in population, it would be beneficial to devote more resources to researching new therapeutic drugs in TCM.

## Figures and Tables

**Table 1:** TCM the protection of islet *β* cells.

TCM	Functions	Genes/proteins	References
Puerarin	Promotes *β* cell proliferation	PDX-1↑	[21]
	Reduces islet OS damage	MDA↓, activity of SOD↑, PGE2, ET, H_2_O_2_, NO levels↓, ICAM-1, NOX2, NOX4 mRNA, and protein expression↓	[41]
Oleanolic acid	Reduces islet OS damage	*α*-glucosidase activity↓	[37]
Ginsenoside Rg3	Reduces *β* cell apoptosis	Phosphorylation of ERK and p38MAPK↑, P44/42MAPK↓	[30]
Rg1		SIL-2R↓, CD4/CD8 and NK cell activity↑, autophagy↑, caspase-39↑, Blc2↑	[9]
Rb1	Reduces islet OS damage	JNK pathway↓, JNK1 and c-Jun↓, IL-6, IL-1*β*, TNF-*α*↓	[46]
Red ginseng	Reduces *β* cell apoptosis	Bcl-2↑, Bax and the activity of caspase-3↓	[31]
	Maintains the integrity of the islet structure	Islet size↑, insulin immunoreactivity↑, islet boundary was irregular, vacuolization↓	
GA	Promotes the synthesis and secretion of insulin	PTP1BmRNA↓, PPAR*γ* genes and PDX-1↑	[9]
	Reduces islet OS damage	ROS↓	[45]
Berberine	Promotes the synthesis and secretion of insulin	The expression of HNF-1*α*/PDX-1↑	[11]
Baicalein	Promotes the synthesis and secretion of insulin	*β* cell membrane K^+^ channel voltage-dependent current↓, intracellular Ca^2+^ concentration in *β* cell↓	[12]
	Promotes *β* cell proliferation	Through cAMP-mediated mechanism	[22]
Resveratrol	Induces stem cell differentiation into *β* cells	The pluripotent stem cell (hiPSC) MSUH-001 ⟶ *β* cell	[25]
curcumin	Reduces islet OS damage	TLR-4↓, caspase-8↓, GM-CSF, G-CSF, IL-6↓, T cell, NK cell↓	[49], [50]
Resveratrol and curcumin	Promotes the synthesis and secretion of insulin	Regulate the expression and activity of PDEs, cAMP level↑	[13]
POP I	Promotes *β* cell proliferation	JNK↑, PDX-1, PPAR*γ*, ERK↑	[20]
	Reduces islet OS damage	JNK1, P38, ERK, and RAS proteins in the JNK pathway↓	
CPOP	Reduces islet OS damage	MDA content and SOD activity↓	[47]
Salidroside	Promotes *β* cell proliferation	Insulin, Pdx-1, GLP-1R mRNA↑IL-1*β* leve↓	[42]
	Reduces OS damage	TXNIP m RNA↓, TRX m RNA↑, ROS↓	[42]
TWP	Induces stem cell differentiation into *β* cells	Stem cell ⟶ the cell which expression of *β* cell functional proteins	[24]
SC2		Stem cell ⟶ new insulin-secreting cells	[26]
Artemisinin	Inducts islet *α* cells into *β* cells	GABA signaling↑, ARX transport from the nucleus to the cytoplasm↓, ARX from chromatin↓, *α* cell secreting glucagon↓, *α* ⟶ *β* cell	[29]
PNS	Reduces *β* cell apoptosis	miR-181b, caspase-3, Bcl-2↑, activation of PI3K-AKT-mTOR pathway↑, Beclin1, LC3-II↓	[32]
cortex mori radicis		PI3K/Akt↑, caspase-3↓, Bcl-2↑, Bax↓	[33]
*Ophiopogonis radix*		NF-kB↓	[34]
CS	Reduces islet OS damage	NO production↓, ROS activity↓, Ca^2+^ concentration↓, UCP-2↑	[40]
TFLB		SOD, GSH↑; MDA↓	[36]
*Anoectochilus roxburghii*		DPPH, ABTS↓, NO↓; iNOS, COX-2, IL-1β↓	[43]
Cinnamaldehyde		The antioxidant enzyme activity↑, free radicals↓	[44]
Mogroside		ROS↓GLUT-2, pyruvate kinase gene↑, JNK signal transduction pathway↓FOXO1↓	[48]
*Polygonatum odoratum*		IFN-*γ*, IL-4↓inflammatory cell infiltration↓, the polarization of Th1 cell↓	[51]
Emodin	Promotes GLP-1 secretion	PPAR*δ*, GLP-1↑	[53]
Trichosanthin	Cell transplantation protection	Slow the rejection and protect the transplanted β cells	[55]
*Euonymus fortunei*		Cell survival rate↑, ROS induced islet cell damage before transplantation↓	[56]
GEB	Improving insulin resistance	Hypothalamic insulin signaling↑, systemic insulin sensitivity↑	[58]

## Abbreviations

ABTS: 2,2ʹ-Azino-bis (3-ethylbenzothiazoline-6-sulfonic acid)
ATP: Adenosine triphosphate
ATG: Arctigenin
Ber: Berberine
CAT: Catalase
cAMP: Cyclic adenosine monophosphate
Caspase: Cysteinyl aspartate specific proteinase
C-Blc2: B-cell lymphoma-2
COX-2: Cyclooxygenase-2
CPOP: Crude *Portulaca oleracea* L. polysaccharide
CHM: Chinese herbal medicines
CS: *Cordyceps sinensis*
DPPH: 1,1-diphenyl-2-picrylhydrazyl
ET: Endothelin
ERK: Extracellular regulated protein kinases
Fox 01: Forkhead box protein 01
FYGL: Ganoderma lucidum polysaccharide
GM-CSF: Granulocyte-macrophage-colony-stimulating factor
GSH-Px: Glutathione peroxidase
GLP-1: Glucagon-like peptide1
GAS: Gastrodin
GEB: Gastrodia elata Blume water extract
GLE: Guava leaf extract
GSIS: Glucose-stimulated insulin release test
GA: Guavenoic acid
GLUT4: Glucose transporter 4
GABA: *γ*-Aminobutyric acid
GSH-Px: Glutathione peroxidase
GSH: Glutathione
HESc: Hydroethanolic extract of *S. cumini* leaf
HG: High glucose
ICAM-1: Intercellular cell adhesion molecule-1
JQJT: JinQi Jiang Tang
JLD: Jin Li Da
JQ-R: Refined-JQ
MAPK: Mitogen-activated protein kinases
MDA: Malondialdehyde
Myrtaceae: *Syzygium cumini* (L.) skeels
NF-*k*B: Nuclear factor-*k*b
NOX2: Nicotinamide adenine dinucleotide phosphate
PKB: Protein kinase B
PTP1B: Protein tyrosine phosphatase-1B
PPAR*γ*: Peroxisome proliferator-activated receptors
PDX-1: Pancreatic duodenal homeobox-1
PDEs: Phosphodiesterases
POP I: *Portulaca oleracea* L.
PNS: Panax notoginseng saponins
PTEN: Phosphatase and tensin homolog deleted on chromosome
PGE2: Tenprostaglandin2
ROS: Reactive oxygen species
RNS: Reactive nitrogen species
SOD: Superoxide dismutase
SST: Somatostatin
STZ: Streptozotocin
SC2: *Syzygium cumini* (L.) skeels
SIL-2R: Soluble interleukin-2 receptor
TCM: Traditional Chinese medicine
TWP: *Tripterygium wilfordii* polysaccharide
TFLB: Total flavonoids of *Lycium barbarum*
TMXKP: Tianmai Xiaoke Pian
TML: Tangminling pill
TXNIP: Thioredoxin-interacting protein
TRX: Thioredoxin
TNF-*α*: Tumor necrosis factor
TLR-4: Toll-like receptors
UCP2: Uncoupling protein 2
XKP: Xiaokeping.
